# Mechanisms of ER Protein Import

**DOI:** 10.3390/ijms23105315

**Published:** 2022-05-10

**Authors:** Sven Lang, Richard Zimmermann

**Affiliations:** Medical Biochemistry and Molecular Biology, Saarland University, 66421 Homburg, Germany

Protein import into the endoplasmic reticulum (ER) is the first step in the biogenesis of approximately 10,000 different soluble and membrane proteins of human cells, which amounts to about 30% of the proteome. Most of these proteins fulfill their functions either in the membrane or lumen of the ER plus the nuclear envelope, in one of the organelles of the pathways for endo- and exocytosis (ERGIC, Golgi apparatus, endosome, lysosome, trafficking vesicles), or at the cell surface as the plasma membrane or secreted proteins. In addition, an increasing number of membrane proteins destined for lipid droplets, peroxisomes or mitochondria are observed to be first targeted to and inserted into the ER membrane prior to their integration into budding lipid droplets or peroxisomes or prior to their delivery to mitochondria via the ER-SURF pathway. ER protein import involves two stages, ER targeting, which guarantees organelle specificity, and insertion of nascent membrane proteins into or translocation of soluble precursor polypeptides across the ER membrane. In most cases, both processes depend on amino-terminal signal peptides or transmembrane helices, which serve as targeting equivalents. However, the targeting reaction can also involve the ER targeting of specific mRNAs or ribosome-nascent chain complexes. In addition, both processes are facilitated by various sophisticated machineries, which reside in the cytosol and the ER membrane, respectively. Except for resident ER, nuclear and mitochondrial membrane proteins, the mature proteins are delivered to their functional locations by vesicular transport.

In this Special Issue, renowned international experts in this area of cell biology report on their structural and mechanistic insights into various aspects of targeting, insertion, and translocation machineries, such as the signal recognition particle (SRP), its corresponding receptor (SR) and the Sec61 complex. M. Pool [1] provides a timely overview about the different pathways for targeting of soluble and membrane proteins to the ER and the triage that is taking place in the cytosol and guarantees delivery of newly synthesized polypeptides to the correct organelle, folding in the cytosol or degradation by the proteasome. H-.H. Hsieh together with S.-ou Shan [2] describe in their chapter the recent progress in deciphering the molecular mechanisms of the paradigm SRP-dependent targeting pathway and the key role of the cytosolic chaperone NAC in preventing protein mistargeting to the ER. As further reading on this subject, we suggest recent original articles by Jomaa et al. [3] as well as Tirincsi et al. [4]. J. Herrmann and colleagues [5] and B. Schrul and colleagues [6] round up the section on protein targeting to the ER and provide a state of the art view of the ER-SURF pathway and recent insights into the client spectrum of the PEX3/PEX19 pathway to the ER, respectively. Here, we suggest as further reading an article on the targeting of mRNAs and ribosome-nascent-chain complexes to the ER [7]. The next section of the Special Issue deals with the machineries for membrane insertion and translocation of proteins in the ER membrane with special emphasis on the central role of the Sec61 complex. Here, A. Tirincsi et al. [8] provide up to date insights into the connections between the different targeting and translocation/insertion machineries, while P. Bhadra and V. Helms [9] as well as M. Liaci and F. Förster [10] focus on molecular dynamics and structural aspects of the Sec61 complex, respectively. This part of the Special Issue is finished off by S.-j. Jung together with H. Kim [11] and P. Whitley et al. [12], who discuss the current views on the Sec62/Sec63 complex in protein translocation and the biophysics of folding and insertion of transmembrane helices at the ER membrane, respectively. As further reading on the structures and molecular mechanisms of additional ER membrane resident membrane protein invertases, we suggest the excellent recent reviews by Borgese et al. [13], O´Keefe et al. [14], and Hegde and Keenan [15]. Last but not least, small molecule inhibitors and toxins that interfere with ER protein import are discussed by K. Vermeire and colleagues [16], thereby providing a link to human medicine, specifically to the so-called Sec61-channelopathies. This medical aspect is discussed by M. Pool [1] and was recently reviewed by Sicking et al. [17]. Two original articles complete the Special Issue, the one by E. Pauwels et al. [18] on the mode of action of the ER protein import inhibitor cyclotriazadisulfonamide (CADA), and the other one by M. Sicking et al. [19] on the adaption of a novel bimolecular luminescence approach to the analysis of the dynamics of ER membrane components, which are in volved in targeting, translocation and membrane insertion of polypeptides at the ER of living human cells.
